# Bayesian selective scan for robust long-horizon action recognition via reliability-aware discretization

**DOI:** 10.3389/frobt.2026.1786524

**Published:** 2026-08-24

**Authors:** Chunjie Wang

**Affiliations:** School of Computer and Software Engineering, Chengdu Jincheng College, Chengdu, Sichuan, China

**Keywords:** adversarial robustness, bayesian uncertainty, calibration, long-horizon action recognition, memory contamination, persistence, reliability-aware discretization, selective state space models

## Abstract

**Introduction:**

Long-horizon action recognition is increasingly deployed in streaming and safety-critical settings, where short but severe corruptions, such as motion blur or adversarial perturbations, can overwrite temporal memory and cause errors to persist after unreliable segments end. Selective State Space Models (SSMs) enable linear-time inference over long contexts, but deterministic discretization can amplify this memory-contamination failure mode.

**Methods:**

We propose BayesMamba, a reliability-aware selective SSM that integrates Bayesian uncertainty into SSM discretization. BayesMamba uses Bayesian Selective Scan (B-Scan) to estimate per-frame aleatoric and epistemic uncertainty, convert uncertainty into a reliability signal, and modulate both the effective time step and input injection so that state updates are suppressed when observations are unreliable. We evaluate BayesMamba on Kinetics-400 (K400) and Something-Something V2 (SSv2).

**Results:**

Under clean evaluations, BayesMamba achieves comparable or slightly improved performance, with 82.4% top-1 accuracy on K400 and 68.7% on SSv2, compared with 82.1% and 68.2% for a deterministic selective SSM baseline, while retaining similar computational cost. On SSv2 under bursty motion blur, BayesMamba improves robust accuracy from 51.4% to 54.2%; under projected gradient descent (PGD, ε = 4/255, ε = 8/255, and ε = 16/255), it improves robust accuracy from 18.2% to 19.8%. BayesMamba also reduces post-corruption persistence, decreasing Persist(64) from 0.41 to 0.19 and Persist(128) from 0.38 to 0.16. Under distribution shift, it improves calibration, reducing corrupted-set ECE from 0.158 to 0.097 and corrupted-set NLL from 2.34 to 1.66.

**Discussion:**

These results indicate that reliability-aware discretization mitigates memory contamination and improves long-horizon robustness with modest overhead while preserving clean action recognition accuracy.

## 1 Introduction

Action recognition is a core capability of visual understanding, and it is widely applied in scenarios such as surveillance, robotics, and embodied interaction. In these scenarios, models need to understand long-horizon video streams in real time and still give stable predictions without excessive drift under environmental interference (Carreira and Zisserman, 2017; Feichtenhofer et al., 2019; Westphal et al., 2017). Long-horizon scenarios usually have characteristics such as sparse evidence, delayed appearance of key signals, and interfering factors. This brings a contradiction between efficiency and safety: long context requires the model to have scalable inference ability, while safety requires that short-term unreliable observations cannot trigger persistent errors.

In practice, long-horizon inference is usually implemented in an online manner with limited memory. The model processes video frames in temporal order and maintains a compact state that encodes history. This state helps achieve low latency, but when the model over-integrates unreliable observations, it may become the concentration point of failure: even after a short contaminated segment has ended, the internal state may still remain biased, leading to subsequent prediction errors. Standard robustness metrics usually measure accuracy on the whole contaminated video, but cannot characterize this failure mode after the contamination ends, and this problem is key in safe streaming inference.

Selective State Space Models (SSMs) provide efficiency characteristics for long-horizon video modeling, because while maintaining linear-time inference and linear memory cost, they also preserve strong sequence modeling ability through selective scanning (Gu and Mamba, 2023; Gu et al., 2022). This has already been reflected in recent video Mamba and visual SSM architectures (Li et al., 2024; Zhu et al., 2024; Yang et al., 2024). Compared with Transformer, Transformer incurs quadratic attention cost in the number of tokens (Bertasius et al., 2021; Arnab et al., 2021; Tong et al., 2022). SSM backbones have better scalability when the temporal span grows, and can maintain more stable inference latency when videos become longer. It should be pointed out that the temporal scale expansion problem here is different from spatial model scaling such as Efficient Net (Paszke et al., 2019), because the core challenge focused on in this paper is to improve per-frame efficiency while maintaining reliable state evolution over long videos. Because the discretized state update that accumulates long-term evidence may also unreliable evidence. When the discretization parameters are affected by contaminated features, short disturbances may also lead to strong updates and contaminate the memory state.

In real videos, there are often bursty unreliable factors, such as motion blur, occlusion, compression artifacts, and sensor failures (Wang et al., 2016). These disturbances also weaken short-term motion cues, and these cues are usually characterized through correspondence relations similar to optical flow, which makes the model harder to recover after short contamination (Brox et al., 2004). In critical deployment, adversarial threats must also be considered: attackers can construct small perturbations with simple perturbation methods, such as Fast Gradient Sign Method (FGSM) and projected gradient descent (PGD), affecting prediction results, and at the same time may keep the temporal state biased after the perturbation window (Madry et al., 2018; Wei et al., 2019; Hendrycks and Dietterich, 2019; Fan et al., 2021). Based on these problems, we need an update rule with reliability awareness, and it can directly regulate memory updates rather than simply adjusting output confidence.

In recent years, efficient long-sequence modeling has made progress, but there are still two key gaps. First, most temporal backbones determine update strength in a deterministic manner according to input features, and are easily affected by temporally local unreliability; this kind of unreliable information should have its weight reduced in memory updating. Second, *post hoc* calibration methods,such as temperature scaling can recalibrate model confidence, but will not change the dynamics of the internal state, and therefore cannot directly prevent the continuation of errors after the contaminated segment ends.

The question studied in this paper is: under uncertainty-guided discretization, can persistent errors after contamination be reduced and robustness be improved without sacrificing linear-time efficiency? We hypothesize that the signal composed of aleatoric uncertainty and epistemic uncertainty can be integrated into the discretization process of SSM,so that the model automatically adopts a more conservative update strategy when it observes unreliability.

We operationally call this failure mode “memory contamination” and quantify it with a persistence score. For frame-wise predictions ${\left\{\hat{y}\right\}}_{t=1}^{T}$ and a contaminated interval $\left[t_{s},t_{e}\right]$ , we examine the window after the contamination ends, $W=\left\{t_{e}+1,...,\min\left(t_{e}+\tau ,T\right)\right\}$ , and calculate the post-contamination error rate (Equation 1).

$$Persist\left(\tau \right)=\frac{1}{\left|W\right|}\sum_{t\in W}1\left[\hat{y_{t}}\neq y^{⋆}\right].$$ (1)

Lower persistence means that the model recovers faster after the unreliable stage ends. By separately characterizing failures at the level of state dynamics, this metric supplements accuracy on the whole contaminated video; the later qualitative analysis shows the recovery trajectory, and Table 1 explains the robust accuracy under bursty contamination and adversarial attacks.

**TABLE 1:** Robustness under motion blur, FGSM, and PGD on SSv2.

Model	Frames	Clean (%)	Motion blur (%)	FGSM (%)	PGD (%)
TimeSformer (Bertasius et al., 2021)	8	62.0 (±0.4)	45.3 (±1.2)	14.0 (±0.5)	12.5 (±0.4)
TimeSformer (Bertasius et al., 2021)	16	63.1 (±0.4)	46.2 (±1.1)	14.8 (±0.5)	13.1 (±0.4)
Deterministic SSM (Gu and Mamba, 2023)	8	61.5 (±0.3)	44.8 (±0.9)	20.3 (±0.6)	13.9 (±0.5)
Deterministic SSM (Gu and Mamba, 2023)	16	68.2 (±0.2)	51.4 (±0.8)	24.9 (±0.6)	18.2 (±0.5)
Temperature-scaled SSM (Salman et al.,2020)	16	68.2 (±0.2)	51.2 (±0.8)	25.1 (±0.6)	18.5 (±0.5)
BayesMamba (ours)	8	61.9 (±0.2)	47.5 (±0.6)	22.1 (±0.4)	15.6 (±0.3)
BayesMamba (ours)	16	**68.7 (±0.2)**	**54.2 (±0.5)**	**26.5 (±0.4)**	**19.8 (±0.3)**

Bold values indicate the best result among the compared methods in the corresponding column. For accuracy and throughput, higher values are better; for ECE, NLL, latency, and persistence-related metrics, lower values are better.

This paper proposes BayesMamba, a reliability SSM for robust HAR oriented to long horizons. Its core idea is Bayesian Selective Scan (B-Scan): estimating uncertainty frame by frame and using uncertainty to regulate the effective time step and input injection strength in SSM discretization. When motion blur, occlusion, or PGD appears, uncertainty rises, the effective step size shrinks, and state updating becomes conservative, thereby alleviating memory contamination.

The contributions of this paper are mainly three points. First, it connects robustness in selective SSM video backbones with memory contamination, and proposes a persistence-based measurement method to describe the phenomenon of continued errors after contaminated segments in long-horizon scenarios (Gu and Mamba, 2023; Wang et al., 2016). Second, it proposes BayesMamba and B-Scan, a lightweight reliability-aware discretization mechanism, which regulates the time step and input gain in selective scan updating. This lets uncertainty truly participate in internal model updating rather than being a *post hoc* calibration signal (Kendall and Gal, 2017; Gal and Ghahramani, 2016; Lakshminarayanan et al., 2017). Third, evaluation is carried out on Kinetics-400 and Something-Something V2 under bursty motion blur and adversarial attacks (FGSM and PGD), and calibration performance under distribution shift (ECE and NLL) is given. Efficiency and scalability are analyzed, and the corresponding quantitative and qualitative results are summarized in the tables and figures of the paper.

The remainder of this paper is arranged as follows. First, BayesMamba and B-Scan are introduced, including their uncertainty modeling method and training objectives. Then, the experimental protocols for contamination, attacks, and persistence evaluation are described in detail. Finally, the results of the model in accuracy, robustness, calibration, and efficiency are reported, and its significance and limitations are discussed.

## 2 Methods

### 2.1 Overview

**FIGURE 1:**
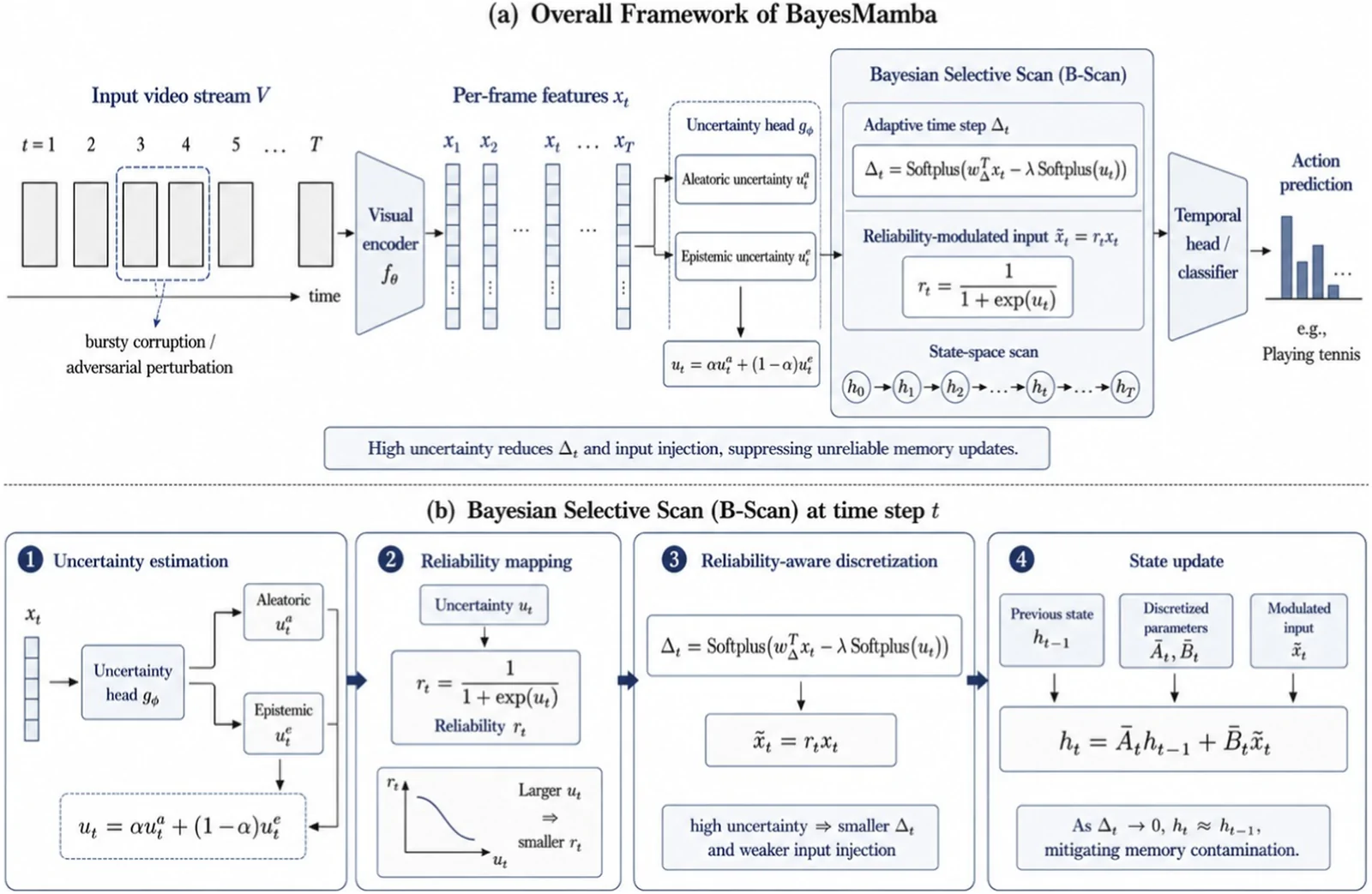
Overview of the BayesMamba framework. **(a)** Overall BayesMamba pipeline: the input video stream may contain bursty corruptions such as motion blur or adversarial attacks; a visual encoder extracts frame-wise features; the uncertainty head estimates aleatoric and epistemic uncertainty; and B-Scan uses the resulting uncertainty signal to modulate the selective scan update for action prediction. **(b)** Detailed Bayesian Selective Scan mechanism: uncertainty estimation, reliability mapping, reliability-aware discretization, and state update jointly suppress unreliable input injection when uncertainty is high.

Figure 1 in the paper illustrates the overall framework of BayesMamba. It processes a video stream V that may contain sporadic disturbances (left side of Figure 1). A standard visual encoder $f_{\theta }$ extracts frame-wise features $x_{t}$ . The core innovation lies in the parallel uncertainty head (center of Figure 1), which simultaneously estimates both aleatoric uncertainty ( $u_{t}^{a}$ ) and epistemic uncertainty ( $u_{t}^{e}$ ) from the features. BayesMamba combines these into a scalar uncertainty signal $u_{t}$ . This uncertainty signal is fed into the B-Scan mechanism (Figure 1, right). By modulating discretization parameters-specifically the time $\Delta_{t}$ and input gain-it dynamically suppresses state updates during unreliable segments.

### 2.2 Problem setting and notation

We considered an input video clip or stream $V=\left\{v_{1},...,v_{T}\right\}$ with *T* frames. A visual encoder $f_{\theta }$ mapped each frame to a feature vector $x_{t}=f_{\theta }\left(v_{t}\right)\in R^{d}$ , where d was the temporal model width. A temporal backbone processed ${\left\{x_{t}\right\}}_{t=1}^{T}$ _1_and produced logits $z\in R^{K}$ over *K* action classes. We assumed streaming inference was supported, so the temporal model maintained a hidden state $h_{t}\in R^{m}$ that was updated online, and the final prediction was produced from an aggregation of states or from the final state.

We focused on two objectives that had to be satisfied simultaneously. Efficiency required that inference scaled linearly in *T* and could be executed with bounded memory, which motivated selective SSM backbones (Gu and Mamba, 2023; Gu et al., 2022). Safety required that state updates were reliability-aware: when $v_{t}$ was corrupted or attacked, the update was suppressed so that the internal memory remained consistent with reliable evidence.

### 2.3 Corruption and threat model

We considered temporally localized corruptions that affected contiguous segments, which is a realistic pattern for motion blur, occlusion, and short sensor glitches (Wang et al., 2016). Let C be a corruption operator that modified a subsequence ${\left\{v_{t}\right\}}_{t=t_{s}}^{t_{e}}$ and produced a corrupted stream *V*. We also considered adversarial perturbations under an $l_{\infty }$ budget. For a given clip, an adversary selected a perturbation $\delta_{t}$ per frame with ${\left\|\delta \right\|}_{\infty }\leq \in$ and optimized the classification loss, resulting in perturbed frames ${\tilde{v}}_{t}=v_{t}+\delta_{t}$ . Fast Gradient Sign Method (FGSM) applied a single gradient step, while projected gradient descent (PGD) applied multiple projected steps and was a stronger iterative stress test (Madry et al., 2018; Hendrycks and Dietterich, 2019).

The key challenge in long horizons is not only that the corrupted frames are misclassified, butalso that the corrupted features can update the internal state and bias subsequent predictions. BayesMamba targets this mechanism by treating uncertainty as a reliability signal that modulates the discretization rule. This design is compatible with standard robustness practices, and it can be combined with adversarial training or consistency regularization in ablations.

### 2.4 Review: selective state space models

An SSM can be expressed as a continuous-time linear dynamical system (Equation 2). Equation 3 gives the corresponding discrete zero-order-hold form used by the selective scan update.

$$\frac{dh\left(t\right)}{dt}=Ah\left(t\right)+Bx\left(t\right),y\left(t\right)=Ch\left(t\right)$$ (2)

Where $A\in R^{m\times m}$ controls state dynamics and *B*,*C* are projection matrices. For discrete-time inference we discretize time with step $\Delta_{t}$ and obtain

$$h_{t}=\bar{A_{t}}h_{t-1}+\bar{B_{t}}x_{t},y_{t}=Ch_{t}$$ (3)

Using a zero-order hold discretization, the discrete parameters satisfy

$$\bar{A_{t}}=\exp\left(\Delta_{t}A\right),\bar{B_{t}}={\left(\Delta_{t}A\right)}^{-1}\left(\exp\left(\Delta_{t}A\right)-I\right)\Delta_{t}B_{t}$$ (4)

Selective SSMs make $\Delta_{t}$ and $B_{t}$ content dependent, typically by projecting $x_{t}$ through small networks and applying element-wise nonlinearities to ensure positivity and stability (Gu and Mamba, 2023; Gu et al., 2022). This selective mechanism improves modeling power on long sequences while retaining linear-time inference.

The standard formulation is deterministic: corrupted features can still produce large $\Delta_{t}$ or large input gain and can inject unreliable evidence into $h_{t}$ . In long horizons, this can create persistence: even if the next frames are clean, the contaminated state can take many steps to recover. This issue is a dynamical analogue of error accumulation, and it may become more severe when corruption is bursty and when the update is highly selective.

### 2.5 Uncertainty modeling

BayesMamba augmented a selective SSM backbone with a lightweight uncertainty head that predicted per-frame aleatoric and epistemic uncertainty. The uncertainty head was implemented as a small projection $g_{\phi }$ operating on $x_{t}$ . Equations 5, 6 define the aleatoric and epistemic uncertainty components estimated bythe uncertainty head.

$$\left(u_{t}^{a},u_{t}^{e}\right)=g_{\phi }\left(x_{t}\right).$$ (5)

Where $u_{t}^{a}$ captures observation noise and $u_{t}^{e}$ captures model uncertainty. We form a scalar uncertainty signal

$$u_{t}=\alpha u_{t}^{a}+\left(1-\alpha \right)u_{t}^{e}$$ (6)

Where α ∈[0,1]is fixed. In practice, blur and compression tend to increase $u_{t}^{a}$ , while adversarial perturbations tend to increase $u_{t}^{e}$ because they push features into regions where the model is less certain (Kendall and Gal, 2017; Lakshminarayanan et al., 2017; Fan et al., 2021).

To obtain epistemic uncertainty, we used stochastic inference via Monte Carlo (MC) dropout on the uncertainty head and a small temporal projection. Given *S* stochastic forward passes, we computed a predictive variance proxy

$$u_{t}^{e}=Var_{s}\left(q_{s}\left(x_{t}\right)\right)$$ (7)

where $q_{s}$ is the stochastic projection for sample s. For aleatoric uncertainty, we predict a logvariance scalar and apply a softplus transformation so the uncertainty is positive:

$$u_{t}^{a}=Softplus\left(r\left(x_{t}\right)\right).$$ (8)

This design followed standard uncertainty estimation practice and Bayesian neural network formulations while keeping additional compute modest in streaming regimes (Kendall and Gal, 2017; Gal and Ghahramani, 2016; Lakshminarayanan et al., 2017; Loshchi et al., 2017). We used a small number of stochastic samples so that inference remained efficient. Table 2 lists the sample count and uncertainty-related hyperparameters, and Table 3 reports additional evaluation on challenging subsets.

**TABLE 2:** Training and model hyperparameters.

Parameter	Value
optimizer	AdamW (Kingma and Ba, 2014; Loshchilov and Hutter, 2019)
epochs	50
base learning rate	3e-4
weight decay	0.05
batch size (global)	128
input resolution	224 × 224
BayesMamba blocks	12
feature dimension *d*	768
MC dropout samples *S*	5
uncertainty head MLP hidden width	384
uncertainty head dropout probability	0.1
uncertainty mix *α*	0.5
uncertainty sensitivity *λ*	1.0
uncertainty regularizer *β*	0.1
smoothness weight *γ*	0.02

**TABLE 3:** Additional evaluation on challenging subsets (Top-1%).

Subset	Deterministic SSM (Gu and Mamba, 2023)	BayesMamba (ours)
SSv2-long compositional	63.2	**64.5**
SSv2 occlusion heavy	49.8	**52.1**
SSv2 compression heavy	53.5	**54.8**
SSv2 lighting drift	55.1	**56.3**

Bold values indicate the best result among the compared methods in the corresponding column. For accuracy and throughput, higher values are better; for ECE, NLL, latency, and persistence-related metrics, lower values are better.

Alternative epistemic estimators are possible. Deep ensembles often produce strong uncertainty but add significant compute and memory in long-horizon settings (Lakshminarayanan et al., 2017). Evidential approaches can yield deterministic uncertainty signals but require careful loss design to avoid miscalibration under shift (Goodfellow et al., 2015). We used Monte Carlo dropout because it was easy to integrate into a streaming pipeline and provided an epistemic signal that was harder to control through a single gradient direction, which was desirable under iterative attacks (Madry et al., 2018; Gal and Ghahramani, 2016).

### 2.6 Bayesian selective scan (B-scan)

Our key principle is that uncertainty should regulate the state update itself. In a discretized SSM, the effective time step $\Delta_{t}$ determines how far the state advances, and the input gain determines how strongly new evidence is injected. If uncertainty is high, both should shrink. Equation 12 defines the stochastic B-Scan state transition used for Monte Carlo paths.

#### 2.6.1 Uncertainty-modulated time step

We predict a base step from features and apply an uncertainty penalty:

$$\Delta_{t}=Softplus\left(w_{\Delta }^{T}x_{t}-\lambda Softplus\left(u_{t}\right)\right).$$ (9)

Where $\Delta_{t}$ represents the effective discretized time-step parameter at frame t , projected from the input frame feature $x_{t}\in R$ using learned weight vector $w_{\Delta }$ ,and modulated by the temporal combined uncertainty metric $u_{t}$ with sensitivity coefficient *λ* > 0 controls sensitivity. When $u_{t}$ increases, $\Delta_{t}$ decreases. In the limit Δ_*t*_→0, the update approaches $h_{t}\approx h_{t-1}$ *because* $A_{t}$ →I and $B_{t}$ →0, so memory is preserved.

#### 2.6.2 Reliability-modulated input

We converted uncertainty into a scalar reliability factor

$$r_{t}=\frac{1}{1+\exp\left(u_{t}\right)}.$$ (10)

Equation 10 makes the reliability mapping explicit: larger uncertainty yields a smaller reliability factor $r_{t}\in$ (0,1], which directly ceases or attenuates input injection. We then dampened the injected evidence via $\tilde{x_{t}}=r_{t}x_{t}$ , where $\tilde{x_{t}}$ represents the reliability-modulated feature. This form ensured that even when the state transition was not fully frozen, unreliable evidence was injected with a smaller gain. The final B-Scan update in Equation 11 was

$$h_{t}=\bar{A_{t}}h_{t-1}+\bar{B_{t}}\tilde{x_{t}}$$ (11)

**FIGURE 2:**
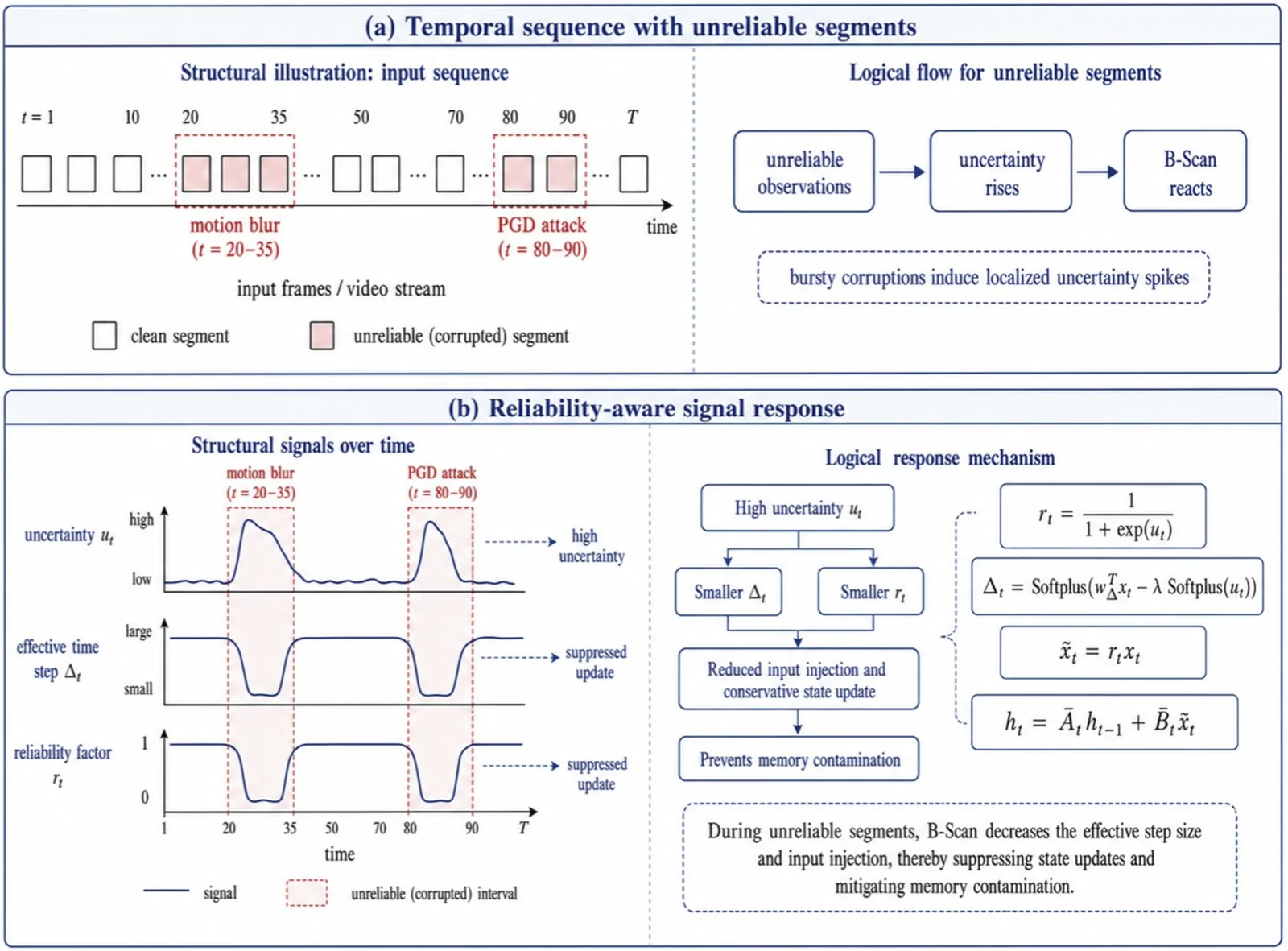
Visualization of reliability-aware discretized signals. **(a)** The time series shows sporadic unreliable segments caused by motion blur (*t* = 20–35) and PGD attacks (*t* = 80–90). A concise summary is provided, showing that unreliable observations increase uncertainty and trigger a B-scan. **(b)** The corresponding signal response indicates that the uncertainty trajectory $u_{t}$ exhibits spikes within these unreliable intervals. Both the effective time step $\Delta_{t}$ and the input reliability $r_{t}$ decrease. The logical mechanism on the right summarizes the reliability-aware updates defined by Equations 9–11.

Where $h_{t}\in R^{d}$ is the recurrent latent state vector of the selective state-space model at time step t. $\bar{A_{t}}=\exp\left(\Delta_{t}A\right)$ and $\bar{B_{t}}={\left(\Delta_{t}A\right)}^{-1}\left(\exp\left(\Delta_{t}A\right)-I\right)\Delta_{t}B_{t}$ are the discretized state transition and input matrices computed from discretization parameters as in Equation 4. We visualized uncertainty and discretization signals over time in Figure 2. Panel (a) presents a schematic temporal sequence with bursty unreliable segments caused by motion blur (*t* = 20–35) and PGD attack (*t* = 80–90), together with a compact logical summary that unreliable observations cause uncertainty to rise and trigger B-Scan. Panel (b) shows the corresponding signal response: the uncertainty trace*u*_*t*_spikes during these unreliable intervals, while both the effective time step $\Delta_{t}$ and the reliability factor *r*_*t*_ decrease. The logical mechanism at the right summarizes the same update pathway defined by Equations 9–11, confirming that B-Scan suppresses unreliable state updates and mitigates memory contamination.

#### 2.6.3 Stability and interpretability

This design is straightforward. Time-step modulation exhibits clear behavior, and the reliability-modulated input injection has easily diagnosable monotonic effects. In practice, this interpretability aids in tuning λ and α The trajectories of $\Delta_{t}$ and $u_{t}$ can be directly inspected as shown in Figure 2. Consequently, the model performs strong updates in reliable segments and tends to be conservative in unreliable segments, and this behavior is consistent across different types of corruption.

#### 2.6.4 Explanation of selective updates

B-Scan can be interpreted as a reliability-aware discretization rule. When uncertainty is low, BayesMamba behaves similarly to a deterministic selective SSM, capable of efficiently learning long-range dependencies (Gu and Mamba, 2023; Gu et al., 2022). When uncertainty is high, the model acts like a stable filter, relying on accumulated memory rather than incorporating unreliable observations. Short-term observations should not overwrite long-term evidence and may even reduce slow drift effects that would otherwise accumulate gradually; thus, this behavior is ideal in burst-corruption scenarios.

#### 2.6.5 Vectorized modulation

In practice, selective scan implementations use vector-valued steps and input gains, which allow different channels of the state to evolve at different effective time scales (Gu and Mamba, 2023; Gu et al., 2022). BayesMamba is compatible with this setting by applying the uncertainty penalty as a shared reliability factor that multiplies the pre-activation for $\Delta_{t}$ and that dampens the input uniformly. This shared factor is sufficient to control memory contamination because contamination is induced by unreliable observations that affect the feature vector broadly. In ablations, applying the reliability factor to only a subset of channels yields weaker robustness because contamination can still enter through unregulated channels.

#### 2.6.6 Relation to gating

B-Scan is not a separate gating network added to the backbone, because the modulation is applied directly to the discretization parameters. This distinction matters in long horizons because the discretization defines the dynamical system that propagates information forward. From this perspective, uncertainty is used to tune the dynamics, not only to mask inputs. The resulting mechanism is easy to diagnose: when $u_{t}$ spikes, both $\Delta_{t}$ and $\tilde{x_{t}}$ shrink, which can be verified by inspecting trajectories as in Figure 2.

### 2.7 Training objective and uncertainty propagation

A key scientific challenge in BayesMamba is ensuring that predicted uncertainties are tightly constrained by the end-task performance without collapsing to degenerate trivial states. In traditional static networks, this typically requires an *ad hoc* output-level heteroscedastic loss to map logits to variance. In contrast, BayesMamba propagates the uncertainty head $g_{\phi }$ directly into the state transition dynamics of the state space model. Consequently, the uncertainty signal is constrained and trained end-to-end via standard task objectives through the recurrence.

#### 2.7.1 Stochastic optimization and variational approximation

During training, we perform stochastic inference over the variational distribution of network weights $W^{s}∼q\left(W\right)$ using active MC dropout inside the uncertainty head $g_{\phi }$ . For each training step, we sample S independent weight realizations, leading to S stochastically updated memory states:

$$h_{t}^{s}=\bar{A}\left(u_{t}^{s}\left(W^{s}\right)\right)h_{t-1}^{s}+\bar{B}\left(u_{t}^{s}\left(W^{s}\right)\right){\tilde{x}}_{t}^{s},s=1,...,S$$ (12)

where $h_{t}^{s}\in R^{d}$ is the stochastically propagated state vector for the *s*th Monte Carlo path, updated with the discretized dynamics $\bar{A}\left(u_{t}^{s}\left(W^{s}\right)\right)h_{t-1}^{s}$ and $\bar{B}\left(u_{t}^{s}\left(W^{s}\right)\right)$ , $u_{t}^{s}\left(W^{s}\right)$ is the combined uncertainty sampled at step t under the *s*th weight realization, and $\bar{x_{t}^{s}}=r_{t}^{s}\left(W^{s}\right)x_{t}$ is the reliability-modulated input features for path s under reliability factor rs. The final predicted class distribution represents the Monte Carlo ensemble average over these stochastic paths as shown in Equation 13:

$$\bar{p}\left(y\left|V\right.\right)=\frac{1}{S}\sum_{S=1}^{S}p\left(y\left|h_{T}^{s}\right.\right)$$ (13)

where $p\left(y^{⋆}\left|h_{T}^{s}\right.\right)$ represents the probability distribution predicted by applying a linear classifier head followed by a softmax function on top of the final temporal state $h_{T}^{s}$ of path s. Our classification objective is the cross-entropy loss over this ensemble prediction in Equation 14:

$$L_{CE}=-\log\tilde{p}\left(y^{⋆}\left|V\right.\right)=-\log\left(\frac{1}{S}\sum_{S=1}^{S}p\left(y^{⋆}\left|h_{T}^{s}\right.\right)\right).$$ (14)

where $y^{⋆}$ denotes the ground-truth action label of the input video sequence $V={\left\{v_{t}\right\}}_{t=1}^{T}$ .

#### 2.7.2 The gradient gating mechanism (backpropagation constraints)

The gradients of $L_{CE}$ with respect to the uncertainty head parameters ϕ flow backward through the entire temporal recurrence of B-Scan as shown in Equation 15:

$$\frac{\partial L_{CE}}{\partial \phi }=\sum_{t}^{T}\frac{\partial L_{CE}}{\partial h_{t}^{s}}\frac{\partial h_{t}^{s}}{\partial u_{t}^{s}}\frac{\partial u_{t}^{s}}{\partial \phi }$$ (15)

This end-to-end backpropagation mathematically constrains and regulates $u_{t}$ through a two-sided constraint:

1. Anti-over-conservatism (Lower Bound): If the model falsely predicts high uncertainty $u_{t}^{s}$ on clean, highly informative frames, B-Scan shrinks the discretization step ( $\Delta_{t}^{s}$ → 0 in Equation 9) and input gain ( $r_{t}^{s}$ → 0 in Equation 10), freezing the state updates. Consequently, the final state $h_{T}^{s}$ lacks needed temporal information, causing cross-entropy $L_{CE}$ to rise. Thus, $L_{CE}$ forces $u_{t}^{s}$ → 0 for clean, reliable inputs to ensure maximum task-relevant information flow.
2. Anti-contamination (Upper Bound): If the model encounters corrupted frames (e.g., motion blur, compression artifacts, adversarial inputs) that deviate from the true video distribution, updating the memory state $h_{t}^{s}$ with these outliers would contaminate the temporal memory, degrading subsequent predictions and increasing $L_{CE}$ . This task-performance loss rewards predicting high uncertainty $u_{t}^{s}$ on corrupted frames because freezing the state update shields the memory from contamination.

#### 2.7.3 Analytical KL divergence regularization

To prevent degenerate solutions where uncertainty values grow unchecked or collapse during early training phases, we incorporate an analytical KL-divergence regularizer. We formalize the predicted uncertainty $u_{t}$ as representing a temporal posterior over the reliability state $q\left(z_{t}\right)=N\left({u_{t}^{a},\left(u_{t}^{e}\right)}^{2}\right)$ , where the aleatoric uncertainty $u_{t}^{a}$ parameterizes the mean estimated observation noise, and the epistemic uncertainty $u_{t}^{e}$ represents the variance proxy under dropout weight sampling. We regularize this posterior toward a zero-centered isotropic prior $\pi_{0}=N\left(0,\sigma_{0}^{2}\right)$ , which penalizes unnecessary gating. Equations 17, 18 define the temporal smoothness term and the full regularized training objective.

$$L_{KL}=\frac{1}{T}\sum_{t=1}^{T}KL\left(q\left(z_{t}\right)\left\|\pi_{0}\right.\right)=\frac{1}{2T}\sum_{t=1}^{T}\left[\frac{{\left(u_{t}^{a}\right)}^{2}+{\left(u_{t}^{e}\right)}^{2}}{\sigma_{0}^{2}}-1-\ln\frac{{\left(u_{t}^{e}\right)}^{2}}{\sigma_{0}^{2}}\right].$$ (16)

Equation 16 explicitly defines the KL regularizer that discourages uniformly inflated uncertainty estimates across time, where $\sigma_{0}^{2}$ represents the prior Gaussian variance parameter (set to a constant $\sigma_{0}^{2}$ = 1.0).

The full loss is:

$$L=L_{CE}+\beta L_{KL}+\gamma L_{smooth}.$$ (17)

where $L_{smooth}=\frac{1}{T-1}\sum_{t=2}^{T}\left|u_{t}-u_{t-1}\right|$ is a temporal smoothness term constraining consecutive frame uncertainty variations. β and γ represent the weighting hyperparameters controlling the strength of the KL regularizer and the temporal smoothness constraint, respectively. This regularization is motivated by calibrated uncertainty estimation under shift and improves stability in streaming inference (Kendall and Gal, 2017; Salman et al., 2020; Guo et al., 2017).

We additionally monitored whether uncertainty was correlated with errors, because a gating mechanism was only meaningful when uncertainty predicted unreliability. In the experiments, we therefore report calibration metrics as well as qualitative uncertainty traces. Equations 17, 18 define the temporal smoothness term and the full regularizedtraining objective.

### 2.8 Robustness protocol in training

We primarily evaluated robustness, and the core method did not require adversarial training. However, we optionally added a lightweight consistency loss under corruption for improved stability. We created a corrupted clip $\tilde{V}$ by applying a corruption operator to a contiguous segment and encouraged predictions to be consistent:

$$L_{cons}=KL\left(p\left(\cdot \left|V\right.\right)\left|\right|\left\|p\left(\cdot \left|\tilde{V}\right.\right)\right.\right).$$ (18)

where $L_{cons}$ is the consistency loss, regulating the model’s predictions on a clean clip V and its corrupted counterpart $\tilde{V}$ . This term was small and was used only in selected ablations. It was included to test whether uncertainty-aware updates interacted favorably with standard robustness regularization (Wang et al., 2016; Fan et al., 2021).

For adversarial robustness, we did not rely on adversarial training in the main setting because it was costly and because our goal was to test whether reliability-aware updates provided robustness without changing the backbone family. We nevertheless included an additional ablation that applied adversarial training with PGD on a subset.

### 2.9 Complexity

BayesMamba preserved linear-time complexity in T because B-Scan required only per-frame projections and element-wise operations. The uncertainty head was computed once per frame, and Monte Carlo dropout used a small number of samples. The overall cost remained dominated by the visual encoder and the SSM backbone. Later efficiency experiments report throughput, latency, and scaling behavior for the full system.

### 2.10 Algorithmic summary

We summarized BayesMamba as an explicit streaming procedure. For each incoming frame,we computed a feature vector, predicted uncertainty, mapped uncertainty to a reliability signal, and then updated the SSM state using B-Scan. The algorithm was simple enough to be implemented as a drop-in modification of a deterministic selective scan, and it did not require revisiting past frames.

Algorithm 1 described the update. We initialized the state $h_{0}$ to zero and maintained it across time. For each t, we computed $x_{t}$ from the visual encoder, estimated $\left(u_{t}^{a},u_{t}^{e}\right)$ with alightweight head, and computed the combined uncertainty $u_{t}$ . We then computed a base selective step and applied the uncertainty penalty to obtain $\Delta_{t}$ . Finally, we computed the reliability factor $r_{t}$ and updated the state. Because each step used only current features and the previous state, inference was streaming and linear time.

Algorithm 1 BayesMamba streaming update.

**Input:** frames ${\left\{v_{t}\right\}}_{t=1}^{T}$ ; visual encoder parameters θ ; uncertainty head parameters ϕ ; time-step projection $w_{\Delta }$ ; uncertainty sensitivity λ; uncertainty mix α (optional) MC dropout samples S.
**Output:** logits from a temporal head applied to the resulting states (e.g., final-state or pooled-state logits). Initializ $h_{0}\leftarrow 0$ .
For t = 1 to T:
Compute $x_{t}=f_{\theta }\left(v_{t}\right).$

Compute $\left(u_{t}^{a},u_{t}^{e}\right)\leftarrow g_{\phi }\left(x_{t}\right)$ with stochastic sampling (e.g., MC dropout withSpasses).
Compute $u_{t}\leftarrow \alpha u_{t}^{a}+\left(1-\alpha \right)u_{t}^{e}.$

Compute $\Delta_{t}\leftarrow Softplus\left(w_{\Delta }^{T}x_{t}-\lambda Softplus\left(u_{t}\right)\right).$

Compute reliability $r_{t}\leftarrow 1/\left(1+\exp\left(u_{t}\right)\right).$

Compute $\tilde{x_{t}}\leftarrow r_{t}x_{t}.$

Compute content-dependent $B_{t}$ from $x_{t}$ (as in selective scan implementations).
Compute $\left(\bar{A_{t}},\bar{B_{t}}\right)$ from $\left(\Delta_{t},A,B_{t}\right)$ via the discretization rule (Equation 4).
Update $h_{t}\leftarrow \bar{A_{t}}h_{t-1}+\bar{B_{t}}\tilde{x_{t}}.$

End for.

Although Algorithm 1 is written in a generic SSM form, it is directly compatible with selective scan implementations that already compute $\bar{A_{t}}$ and $\bar{B_{t}}$ t as functions of $x_{t}$ (Gu and Mamba, 2023; Gu et al., 2022). The only additional operations are the uncertainty projections and the scalar transformations for $\Delta_{t}$ and $\tilde{x_{t}}.$

### 2.11 Memory contamination analysis

We provide a simple analysis that clarifies why uncertainty modulation can mitigate contamination. Consider a linearized update in which $\bar{A_{t}}$ and $\bar{B_{t}}$ vary slowly within a short window, and suppose a corrupted segment produces a feature perturbation $\delta x_{t}$ for t in a contiguous interval.

**FIGURE 3:**
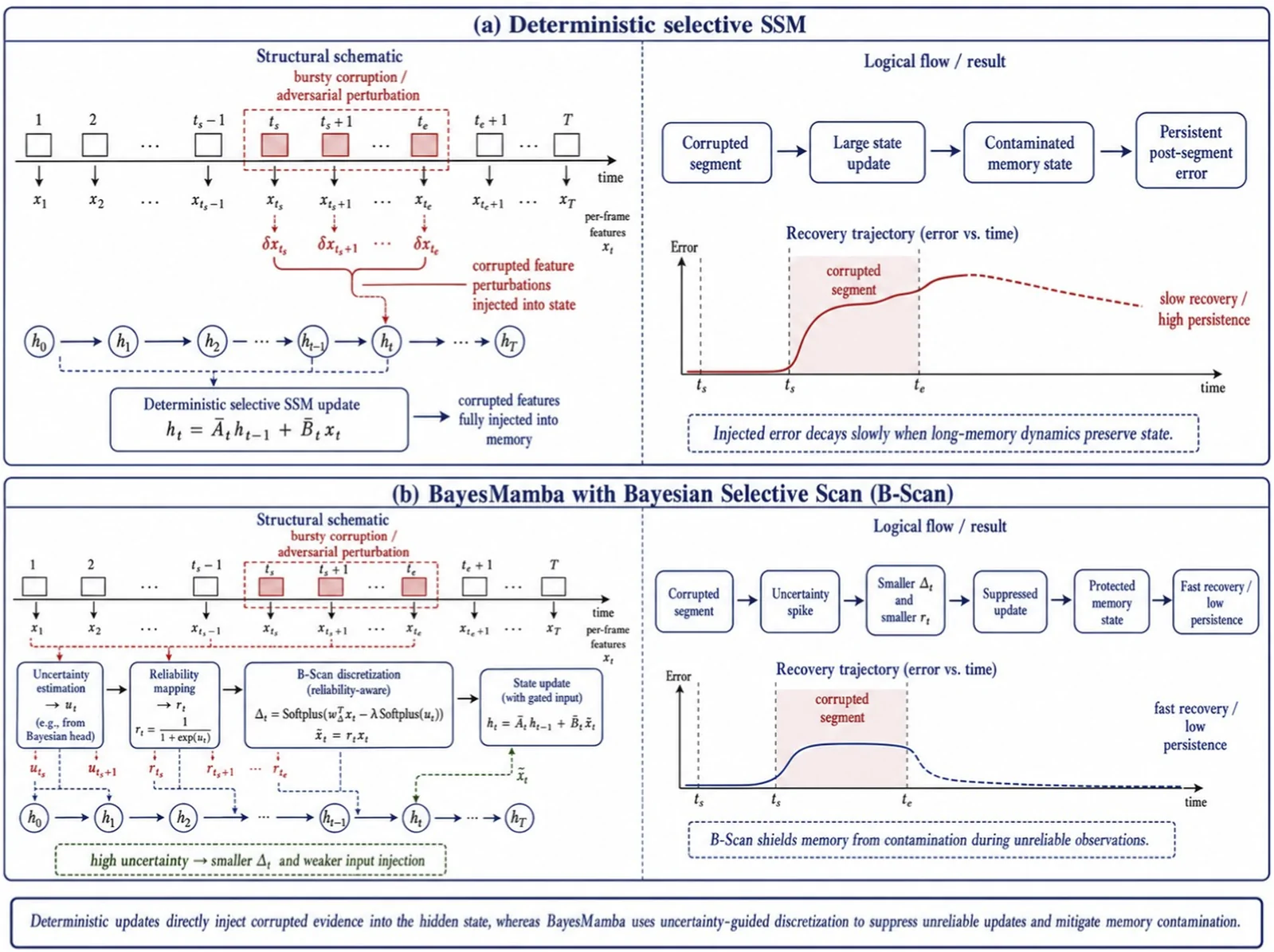
Conceptual comparison of memory contamination mechanisms. **(a)** In a deterministic selective SSM, corrupted features are fully injected into the hidden state, causing errors to persist long after the corruption ends. **(b)** In BayesMamba, B-Scan acts as a reliability gate: high uncertainty shrinks the time step and input gain, thereby shielding the memory state from contamination and accelerating recovery.

Figure 3 conceptually compares the update dynamics. In a deterministic update (Figure 3, top), the perturbation is injected into the state as $\bar{B_{t}}\delta x_{t}$ , and subsequent states contain accumulated contributions of this injection through repeated multiplication by $\bar{A_{t}}$ . If the spectral radius of $\bar{A_{t}}$ is close to one, which is typical for long-memory dynamics, the injected error can decay slowly. This slow decay is desirable for preserving useful evidence, but it is harmful when the injected signal is unreliable.

In BayesMamba (lower half of Figure 3), uncertainty increases during contaminated segments, while temporal modulation reduces $\Delta_{t}$ . This reduction causes $\bar{A_{t}}$ to approach the identity matrix and $\bar{B_{t}}$ to approach zero. The input injection method used in reliability modulation further reduces the amplitude of the injection disturbance. These effects reduce the injected error terms and weaken the ability of brief contaminated segments to overwrite the state. This mechanism is particularly important for sudden corruption, where uncertainty spikes are temporally localized, allowing the model to update normally before and after the burst while remaining conservative during the burst.

The analysis also reveals a clear trade-off. If uncertainty increases at all locations, the model’s updates become conservative, potentially leading to underfitting because the model cannot incorporate new evidence. The KL regularization term and the smoothing term are also crucial, as they prevent degenerate solutions and encourage uncertainty to remain sparse over time.

This aligns with the burst-like characteristics of many real-world corruption scenarios (Kendall and Gal, 2017; Wang et al., 2016; Salman et al., 2020). Subsequent calibration results indicate that BayesMamba maintains good calibration rather than simply uniformly increasing uncertainty across the board.

### 2.12 Practical implementation details

To ensure reproducibility and provide complete technical clarity, we specify the detailed implementation parameters and structural configurations of BayesMamba below.

#### 2.12.1 Uncertainty head layer structure

The uncertainty head $g_{\phi }$ is implemented as a lightweight two-layer Multi-Layer Perceptron (MLP) mapping the per-frame 2D visual feature $x_{t}\in R^{d}$ (where *d* = 768 is the hidden state width) to the uncertainty space. Equations 19–22 specify the Monte Carlo epistemic proxy, aleatoric variance proxy, normalized uncertainty trace, and reliability mapping used in the implementation. Let the middle dimension be *d*_mid_ = 384. The exact forward operations are:

1. First linear layer: $z_{t}=W_{1}x_{t}+b$ , where $W_{1}\in R^{d_{\text{mi}d}\times d}$ and $b_{1}\in R^{d_{mid}}$ .
2. Activation function: $a_{t}=SiLU\left(z_{t}\right).$

3. Dropout layer (active during stochastic sampling): We apply a Dropout layer immediately after the SiLU activation with a dropout probability $p_{drop}=0.1$ .
4. Epistemic head: $q_{t}=W_{e}a_{t}+b_{e}$ , where $W_{e}\in R^{d_{mid}\times d_{mid}}$ and $b_{e}\in R^{d_{mid}}$ projects to a secondary temporal representation. This is sampled S = 5 times during training and testing to compute the epistemic variance proxy (Equation 7):
$$u_{t}^{e}=Var_{s}\left(q_{t}^{s}\right)\in R$$ (19)
  Stochastic weight-based MC dropout remains active in both training and inference phases to guarantee that the epistemic uncertainty dynamically responds to out-of-distribution shifts (such as motion blur or hostile perturbations) during online streaming.
5. Aleatoric head: $r\left(x_{t}\right)=W_{a}a_{t}+b_{a}$ , where $W_{a}\in R^{1\times d_{\text{mi}d}}$ and $b_{a}\in R$ outputs a single scalar. The final positive aleatoric noise variance proxy is computed via softplus (Equation 8):

$$u_{t}^{a}=Softplus\left(r\left(x_{t}\right)\right)\in R$$ (20)

#### 2.12.1 Normalization and reliability parameters

To reduce scale sensitivity across video clips with varying content, we additionally monitor a normalized uncertainty trace derived from the scalar uncertainty signa $u_{t}=\alpha u_{t}^{a}+\left(1-\alpha \right)u_{t}^{e}\left(with\alpha =0.5\right):$

$$\hat{u_{t}}=\frac{u_{t}-\mu_{t}}{\sigma_{t}+\epsilon_{u}}.$$ (21)

where $\mu_{t}$ and $\sigma_{t}$ represent running statistics tracked recursively with a momentum factor of $\rho_{u}=0.9:$

$$\mu_{t}=\rho_{u}\mu_{t-1}+\left(1-\rho_{u}\right)u_{t},\sigma_{t}^{2}=\rho_{u}\sigma_{t-1}^{2}+\left(1-\rho_{u}\right){\left(u_{t}-\mu_{t}\right)}^{2}$$ (22)

and *ϵ*_*u*_ = 10^–5^ prevents division-by-zero. We use this normalized trace for diagnostic visualization and stability monitoring, but the forward B-Scan update itself follows the same reliability mapping as Equation 10, namely, *r*_*t*_ = 1/(1 + exp ( $u_{t}$ )), so that the implementation remains aligned with the abstract mechanism shown in Figure 1.

#### 2.12.2 Parallel training efficiency and selective scan kernel integration

Because selective scans in Mamba require sequential dependency over time ( $h_{t}$ depends on *h*_*t*−1_), parallelizing training across the time dimension is achieved in practice via an associative scan parallel kernel on GPUs (Gu and Mamba, 2023; Gu et al., 2022).

B-Scan Integration: Our reliability-aware discretization parameters $\Delta_{t}$ (Equation 9) and modified inputs $\tilde{x_{t}}=r_{t}x_{t}$ are computed using standard, element-wise tensor broadcasting in PyTorch *prior* to executing the CUDA selective scan kernel. Associative Scan Compatibility: The discretized transition matrix $\bar{A_{t}}$ and step input matrix $\bar{B_{t}}$ in B-Scan are computed with the same zero-order-hold parameterization introduced in Equation 4, i.e., $\bar{A_{t}}=\exp\left(\Delta_{t}A\right)$ , and $\bar{B_{t}}={\left(\Delta_{t}A\right)}^{-1}\left(\exp\left(\Delta_{t}A\right)-I\right)\Delta_{t}B_{t}$ while the reliability-modulated feature $\tilde{x_{t}}=r_{t}x_{t}$ is injected through the standardselective scan update in Equation 11. This preserves the standard associative scan structure used by Mamba-style kernels:

$$\left(h_{t},\bar{A_{t:k}}\right)=\left(h_{k},\bar{A_{k}}\right)\cdot \left(h_{t-1},\bar{A_{t-1}}\right)$$ (23)

where · is the associative binary operator. Equation 23 shows the associative scan form that preserves compatibility with Mamba-style selective scan kernels.

Parallel Speedup: Consequently, the entire B-Scan can be natively solved using the highly optimized parallel associative scan CUDA kernel (Gu and Mamba, 2023) in *O* (log *T*) parallel time. The overhead of computing the lightweight linear projections of the 2-layer MLP uncertainty head adds less than 2.5% execution latency during training, maintaining high hardware utilization and parallel throughput.

## 3 Experiments

### 3.1 Setup

#### 3.1.1 Datasets

We evaluated on Kinetics-400 (K400) (Carreira and Zisserman, 2017; He et al., 2016) and Something-Something V2 (SSv2) (Goyal et al., 2017). K400 covered diverse actions across scenes and supported generalization assessment, while SSv2 emphasized temporal relations and ordering and was sensitive to long-horizon modeling because similar frames could correspond to different actions depending on temporal context. We also constructed an extended-length SSv2-long subset by concatenating adjacent clips with matching actors to simulate longer horizons, following common practice for long-context evaluation (Gu et al., 2022; Tong et al., 2022).

#### 3.1.2 Baselines

We compared BayesMamba to a CNN baseline (SlowFast) (Feichtenhofer et al., 2019), a Transformer baseline (TimeSformer) (Bertasius et al., 2021), and a deterministic selective SSM baseline based on Mamba-style selective scanning adapted to video features (Gu and Mamba, 2023). We also reported a calibration-oriented baseline with temperature scaling on top of the deterministic SSM to separate calibration from update regulation (Salman et al., 2020).

#### 3.1.3 Architectures

For controlled comparison, the SSM-based models (Deterministic SSM, Temperature-scaled SSM, and BayesMamba) used a ResNet-50 visual encoder (Loshchilov and Hutter, 2019), while TimeSformer used its standard ViT-B backbone (Bertasius et al., 2021). For the SSM backbones, we used 12 temporal blocks and width *d* = 768, and we kept parameter counts comparable across variants. BayesMamba added a small uncertainty head and used B-Scan for state updates. The training setup followed standard video recognition practice with AdamW (Kingma and Ba, 2014; Loshchilov and Hutter, 2019) and cosine scheduling (Guo et al., 2022). Hyperparameters are summarized in Table 2.

#### 3.1.4 Training and inference details

Unless otherwise noted, we sampled 16 frames uniformly from each clip for the SSM-based models (Table 4) and used the standard 8-frame protocol for TimeSformer (Bertasius et al., 2021). We used standard video augmentations during training (random resized crop to 256 pixels, random crop to 224 × 224, and random horizontal flip *p* = 0.5) and evaluated with a single-view protocol (center crop of 224 × 224). We initialized the visual encoder frompublicly available pretrained weights (ImageNet-1K supervised (Jia et al., 2009)) rather than large-scale video self-supervised pretraining such as VideoMAE (Tong et al., 2022), and fine-tuned end-to-end. We implemented all training and evaluation code in PyTorch (Nixon et al., 2019). We report the complete training recipe (batch size, resolution, and learning-rate schedule details) in Table 2.

**TABLE 4:** Clean accuracy and compute on K400 and SSv2.

Model	Backbone	Frames	GFLOPs	K400 top-1 (%)	SSv2 top-1 (%)
SlowFast (Feichtenhofer et al., 2019)	R50	8x8	65	75.6 (±0.2)	61.7 (±0.3)
TimeSformer (Bertasius et al., 2021)	ViT-B	8	196	80.7 (±0.2)	62.0 (±0.4)
TimeSformer (Bertasius et al., 2021)	ViT-B	16	392	81.5 (±0.3)	63.1 (±0.4)
Deterministic	R50	8	22.5	79.8 (±0.2)	61.5 (±0.3)
SSM (Gu and Mamba, 2023)					
Deterministic SSM (Gu and Mamba, 2023)	R50	16	45	82.1 (±0.2)	68.2 (±0.2)
BayesMamba (ours)	R50	8	24	80.1 (±0.2)	61.9 (±0.2)
BayesMamba (ours)	R50	16	48	**82.4 (±0.1)**	**68.7 (±0.2)**

Bold values indicate the best result among the compared methods in the corresponding column. For accuracy and throughput, higher values are better; for ECE, NLL, latency, and persistence-related metrics, lower values are better.

#### 3.1.5 Corruption and attack protocols

We evaluated bursty corruption by applying a contiguous motion blur segment covering 30% of frames with a fixed blur severity (linear motion blur kernel of size 15 at 45°), and we also considered occlusion, compression, and lighting shift (Table 5) (Wang et al., 2016). For adversarial robustness, we evaluated FGSM and PGD under an ℓ ∞ budget *ϵ* = 8/255, with PGD using 10 iterations and step size 2/255 following robust evaluation conventions (Madry et al., 2018; Hendrycks and Dietterich, 2019). We crafted perturbations on the input frames and evaluated the full pipeline, including the visual encoder and temporal backbone.

**TABLE 5:** Corruption suite for robustness evaluation.

Corruption	Severity	Temporal pattern	Motivation
motion blur	strong	contiguous 30% segment	fast motion and camera shake
occlusion	medium	random 20% frames	intermittent obstruction
compression	medium	full clip	streaming artifacts
lighting shift	medium	slow drift	day-to-night transition

**TABLE 6:** Additional adversarial settings on SSv2 (Top-1%).

Model	Transfer PGD (%)	Concentrated PGD (%)	Temporal-coupled attack (%)
Deterministic SSM (Gu and Mamba, 2023)	35.0 (±0.6)	15.0 (±0.5)	17.5 (±0.5)
BayesMamba (ours)	**38.5 (±0.4)**	**18.5 (±0.4)**	**19.2 (±0.3)**

Bold values indicate the best result among the compared methods in the corresponding column. For accuracy and throughput, higher values are better; for ECE, NLL, latency, and persistence-related metrics, lower values are better.

For PGD, we applied the perturbation to each frame independently but computed gradients through the full temporal model so that the attack could exploit temporal coupling. For FGSM, we applied a single gradient step at the same budget. We also included a transfer setting where perturbations were crafted on the visual encoder and transferred to the temporal backbone (Wei et al., 2019; Fan et al., 2021).

#### 3.1.6 Metrics

This paper reports top-1 accuracy, robust accuracy under interference and attack conditions, and calibration quality under clean distributions and interfered distributions.

Calibration is measured using Expected Calibration Error (ECE) and negative log likelihood (NLL) (Salman et al., 2020). A persistence score is computed to measure how long predictions continue to remain incorrect after the interfered segment ends. This metric complements the qualitative visualizations of the mechanism in Figures 2, 3. For ECE, predictions are divided into different confidence bins, and the weighted average gap between accuracy and confidence is computed. For NLL, the mean negative log probability assigned to the true label is reported. To characterize variance, 3 random seeds are trained for each model and the mean and standard deviation are reported. Bootstrap resampling across video clips is used to estimate confidence intervals for key robustness comparisons (Efron and Tibshirani, 1993).

### 3.2 Ethics statement

Publicly available benchmark datasets (Kinetics-400 and Something-Something V2) are used, and no new human-subject data are collected. The licenses and terms of use of these datasets are followed (Kinetics-400: Creative Commons Attribution 4.0 International; Something.

Something V2: Research Use Only). These datasets contain human actions, but do not contain personally identifiable information (PII), and no offensive content is targeted or generated. The research focuses on robustness and safety in action recognition, aiming to improve system reliability in assistive robotics and human-computer interaction. However, long-horizon surveillance technology has the risk of being misused for unauthorized monitoring. It is advocated that such systems should strictly follow privacy regulations and ethical guidelines when deployed in real environments. Since this study relies only on public data, institutional review board (IRB) approval is not required.

### 3.3 Main results

Table 4 reports accuracy and computation under clean conditions. In order to conduct fair and comprehensive comparisons across different frame lengths, our method and each baseline model are evaluated under aligned frame configurations (8 frames and 16 frames).

Under 8 frames, BayesMamba reaches 80.1% Top-1 on K400 and 61.9% on SSv2, with 24 GFLOPs of computation. Its performance is comparable to 8-frame TimeSformer (80.7% on K400 and 62.0% on SSv2), while computation cost is reduced by 8.2 times (24 GFLOPs vs. 196 GFLOPs). Under the 16-frame budget, BayesMamba reaches 82.4% Top-1 on K400 and 68.7% on SSv2, with 48 GFLOPs of computation. Its performance is more competitive than 16-frame TimeSformer (81.5% on K400 and 63.1% on SSv2), while GFLOPs usage is reduced by 8.1 times (48 GFLOPs vs. 392 GFLOPs). Compared with the deterministic baseline (82.1% on K400 and 68.2% on SSv2), the improvement in clean accuracy is relatively limited, but these results show that embedding Bayesian uncertainty into the SSM update can maintain comparable performance under normal settings while keeping computational efficiency.

**FIGURE 4:**
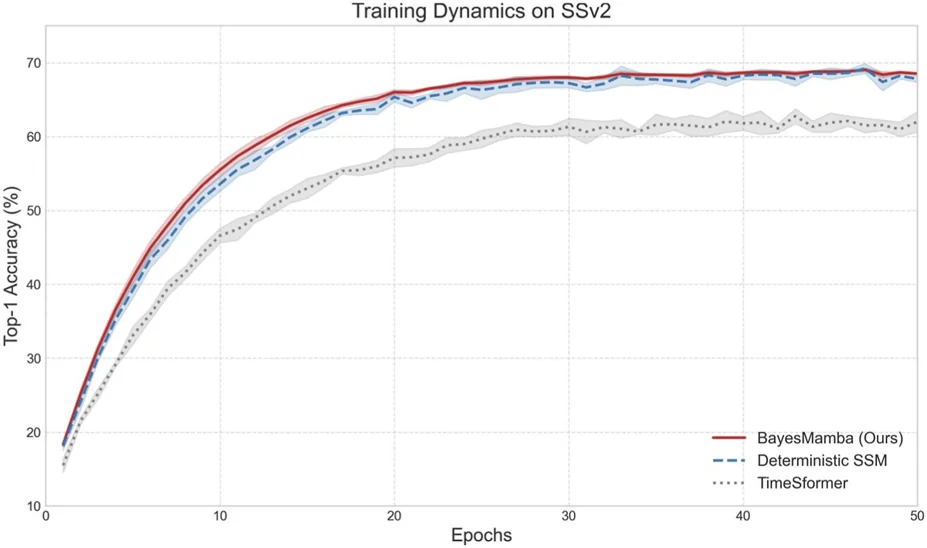
Training dynamics on SSv2 across random seeds.

Figure 4 shows the training dynamics on SSv2. Compared with deterministic SSM and TimeSformer, BayesMamba shows lower variance across different random seeds.

#### 3.3.1 Robustness to motion blur and adversarial attacks

Table 1 summarizes the robustness performance of the models under bursty motion blur and adversarial attacks under aligned frame-length budgets.

When compared under the 8-frame setting, BayesMamba is on par with 8-frame TimeSformer in clean accuracy (61.9% vs. 62.0%), and shows stable robustness improvements under perturbation conditions. It reaches 47.5% under motion blur and 15.6% under PGD, while TimeSformer reaches 45.3% and 12.5%, respectively. Under the 16-frame setting, BayesMamba achieves competitive clean performance of 68.7% (TimeSformer is 63.1%, and the deterministic baseline is 68.2%). Under unreliable conditions it shows stronger robustness (top-1 is 54.2% under motion blur and 19.8% under PGD; by comparison, TimeSformer is 46.2% and 13.1%, and the deterministic baseline is 51.4% and 18.2%, respectively). These results indicate that although the gain in clean performance is small, reliability-modulated state updates bring systematic benefits in corrupted and perturbed feature scenarios where memory protection is crucial.

Persistence is measured. For each video containing a corrupted segment, the prediction results changing over time are tracked. The proportion of frames for which the prediction is still wrong after the corrupted segment ends is recorded.

#### 3.3.2 Persistence evaluation protocol

In order to strictly separate failure modes triggered by state memory contamination, a dedicated Persistence Evaluation Protocol is established as follows:

1. Selection and placement of corrupted segments: For any video clip containing *T* frames, we introduce a transient temporal corruption (for example, motion blur or adversarial PGD noise), which covers a fixed contiguous segment of length $L_{corr}$ = 0.3*T*. This corrupted interval is placed in the middle of the video and is defined as $\left[t_{start},t_{end}\right]$ , where $t_{start}$ = ⌊0.35*T* ⌋ and $t_{end}$ = ⌊0.65*T* ⌋. This setting guarantees a longer clean context before the corrupted segment for memory accumulation, and retains a clean recovery window after it.
2. Online frame-level prediction: Because action recognition backbones usually generate clip-level predictions, frame-level predictions ${\left\{\hat{y_{t}}\right\}}_{t=1}^{T}$ are obtained under online streaming mode. The specific practice is to let the model run progressively over time. At each time step *t*, the recurrent state $h_{t}$ is input to the classification head to obtain the prediction $\hat{y_{t}}\in Y$ . This prediction is based only on the historical context up to time *t*, without leaking future frame information.
3. Parameter *τ* and window selection: The parameter *τ* ∈ {64, 128} determines the length of the post-corruption evaluation window *W* = {*t*_end_ + 1, … , min (*t*_end_ + *τ*, *T*)}. If the length of a video is not enough to cover *t*_end_ + *τ*, then the window is truncated at *T*. In order to avoid boundary artifacts and severe sample-size variance, videos satisfying ∣*W* ∣ < 16 frames are filtered out, to prevent insufficient boundary samples.
4. Category balancing: According to the category-balancing criterion, sampling is conducted from the SSv2 validation subset, with 10 videos randomly selected for each of 174 action categories (1,740 test videos in total). This can maintain balancedrepresentation and avoid bias in average persistence statistics caused by dominant action categories or complex motion categories.
5. Statistical metrics and confidence intervals: By conducting *B* = 10,000 non-parametric bootstrap resampling runs on the video pool, the standard deviations and 95% confidence intervals (CI) of Persist (64) and Persist (128) are computed, thereby ensuring that statistical reliability does not depend on distributional assumptions.

#### 3.3.3 Persistence and recovery

We quantified recovery speed by evaluating Persist (*τ*) over multiple values of *τ* according to the above protocol and by inspecting how quickly error rates returned to the clean baseline after the corrupted segment ended. In a representative SSv2 subset with a fixed 30% blur segment, BayesMamba reduced Persist (64) from 0.41 to 0.19 and reduced Persist (128) from 0.38 to 0.16. Figure 2 visualized the uncertainty, time-step, and reliability signals associated with bursty corruption, while Figure 3 illustrated the corresponding memory-contamination dynamics.

**FIGURE 5:**
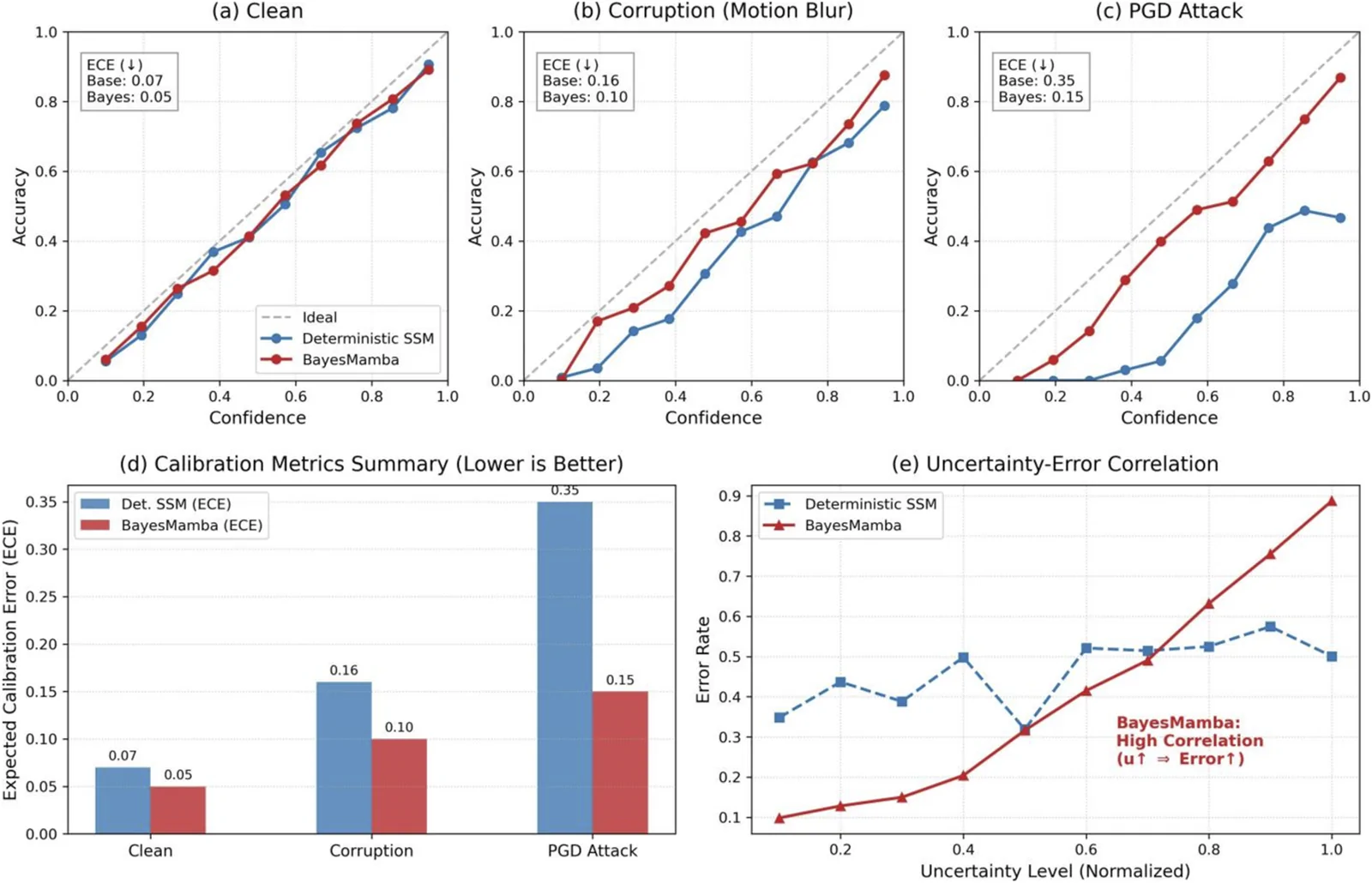
UUncertainty calibration under corruption and adversarial attack. **(a–c)** Reliability diagrams compare calibration under clean, corrupted, and adversarial conditions. **(d)** Expected calibration error summarizes the calibration gap across settings. **(e)** The uncertainty-error relationship shows that BayesMamba’s uncertainty is more closely aligned with prediction error than the deterministic baseline.

Figure 5 reported calibration under distribution shift and attack.

#### 3.3.4 Transfer attacks and temporal coupling

Table 1 reported white-box attacks where gradients were computed through the full pipeline. We also evaluated a transfer threat where perturbations were crafted on the visual encoder alone and then applied to the full model (Wei et al., 2019; Fan et al., 2021). In this setting, BayesMamba remained more robust than deterministic SSMs, with a smaller margin than in the white-box setting.

To test temporal allocation effects, we also considered two PGD variants that differed in how perturbations were allocated across time: (i) a fixed budget applied independently to each frame as in standard evaluation (Madry et al., 2018; Hendrycks and Dietterich, 2019), and (ii) a concentrated perturbation budget applied to a contiguous window. We also examined temporal coupling by optimizing the final clip-level loss under a time-aggregated gradient (Table 7).

**TABLE 7:** Confidence-thresholded update skipping (SSv2).

Model	Clean (%)	Motion blur (%)	PGD (%)	Persist (64)
Update-skip baseline	68.0 (±0.2)	48.0 (±0.9)	15.0 (±0.5)	0.30

#### 3.3.5 Robustness across corruption types

We used motion blur as the primary bursty corruption and also evaluated occlusion, compression artifacts, and gradual lighting drift (Wang et al., 2016). Table 5 summarized the corruption suite, and Table 3 reported results on challenging subsets constructed from each corruption type.

We also analyzed temporal uncertainty responses in Figure 2 and later provide qualitative examples of the aleatoric and epistemic uncertainty behavior.

As an additional baseline, we evaluated a confidence-thresholded update-skipping heuristic (Table 7).

**FIGURE 6:**
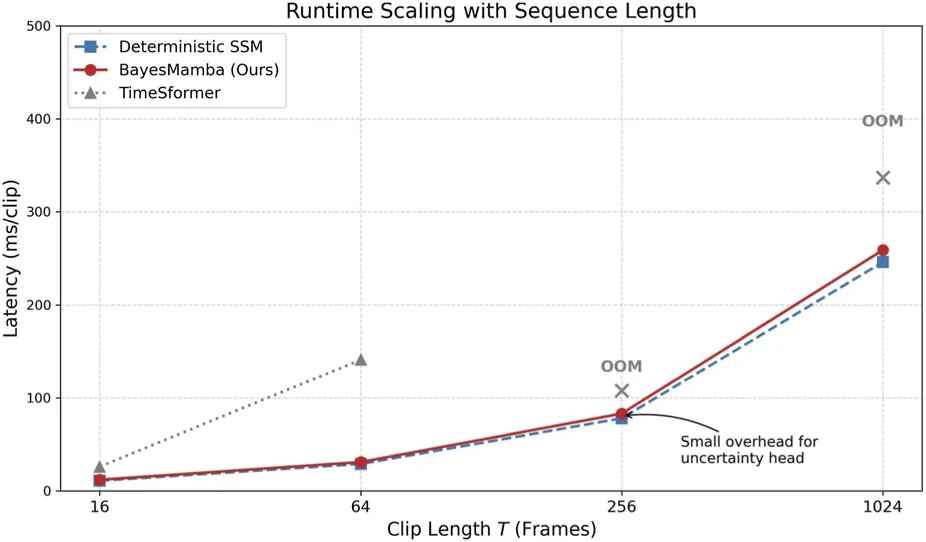
Runtime scaling with clip length for deterministic SSMs and BayesMamba.

We also reported runtime and memory behavior in long-horizon inference (Tables 8, 9; Figure 6).

**TABLE 8:** Efficiency comparison.

Model	Frames	GFLOPs	Throughput (clips/s)	Latency (ms/clip)
TimeSformer (Bertasius et al., 2021)	8	196	38	26
TimeSformer (Bertasius et al., 2021)	16	392	19	52
Deterministic SSM (Gu and Mamba, 2023)	8	22.5	195	6
Deterministic SSM (Gu and Mamba, 2023)	16	45	102	11
BayesMamba (ours)	8	24	185	6.5
BayesMamba (ours)	16	48	96	12

**TABLE 9:** Scaling with clip length on SSv2-long (Top-1%/latency ms).

Model	16 frames	64 frames	256 frames	1,024 frames
TimeSformer (Bertasius et al., 2021)	61.8/26	60.4/141	OOM	OOM
Deterministic SSM (Gu and Mamba, 2023)	67.9/11	67.1/29	65.5/78	63.2/246
BayesMamba (ours)	**69.0/12**	**68.2/31**	**66.8/83**	**65.1/259**

For the 16-frame setting, we evaluate on the standard SSv2 validation set (short clips), which serves as the anchor for the long-video scaling experiments. Bold values indicate the best result among the compared methods in the corresponding column. For accuracy and throughput, higher values are better; for ECE, NLL, latency, and persistence-related metrics, lower values are better.

#### 3.3.6 Ablations

Table 10 reported component ablations.

**TABLE 10:** Component ablation on SSv2.

Variant	Clean (%)	Motion blur (%)	PGD (%)
Deterministic SSM (Gu and Mamba, 2023)	68.2 (±0.2)	51.4 (±0.8)	18.2 (±0.5)
+ reliability-modulated input only	68.3 (±0.2)	52.1 (±0.6)	18.6 (±0.4)
+ uncertainty-modulated Δ_*t*_only	68.4 (±0.2)	53.0 (±0.5)	19.1 (±0.4)
Full BayesMamba	**68.7 (±0.2)**	**54.2 (±0.5)**	**19.8 (±0.3)**

Bold values indicate the best result among the compared methods in the corresponding column. For accuracy and throughput, higher values are better; for ECE, NLL, latency, and persistence-related metrics, lower values are better.

We further studied sensitivity to the uncertainty sensitivity coefficient *λ* and to the aleatoric–epistemic mixing parameter *α* in auxiliary sweeps, and Table 2 reports the final hyperparameter settings used in the main experiments.

We also ablated the stochastic uncertainty estimation by removing Monte Carlo dropout and by varying the number of stochastic samples.

#### 3.3.7 Calibration and uncertainty quality

We evaluated calibration with ECE and NLL on clean and corrupted sets (Salman et al., 2020). Table 11 reports calibration metrics for all models.

**TABLE 11:** Uncertainty calibration metrics (lower is better).

Model	ECEclean	ECEcorrupt	NLLclean	NLLcorrupt
TimeSformer (Bertasius et al., 2021)	0.086	0.142	1.32	2.01
Deterministic SSM (Gu and Mamba, 2023)	0.071	0.158	1.18	2.34
Temperature-scaled SSM (Salman et al., 2020)	0.060	0.141	1.14	2.08
BayesMamba (ours)	**0.054**	**0.097**	**1.12**	**1.66**

Bold values indicate the best result among the compared methods in the corresponding column. For accuracy and throughput, higher values are better; for ECE, NLL, latency, and persistence-related metrics, lower values are better.

We also computed the correlation between uncertainty and error events over time; Figure 5 visualized these temporal patterns.

#### 3.3.8 Efficiency and scaling

We compared compute and latency across clip lengths. Table 8 reports throughput and latency under aligned frame configurations, and Table 9 reports scaling behavior on SSv2-long. Figure 6 visualizes runtime scaling with clip length.

#### 3.3.9 Corruption suite, hyperparameters, and additional results

**FIGURE 7:**
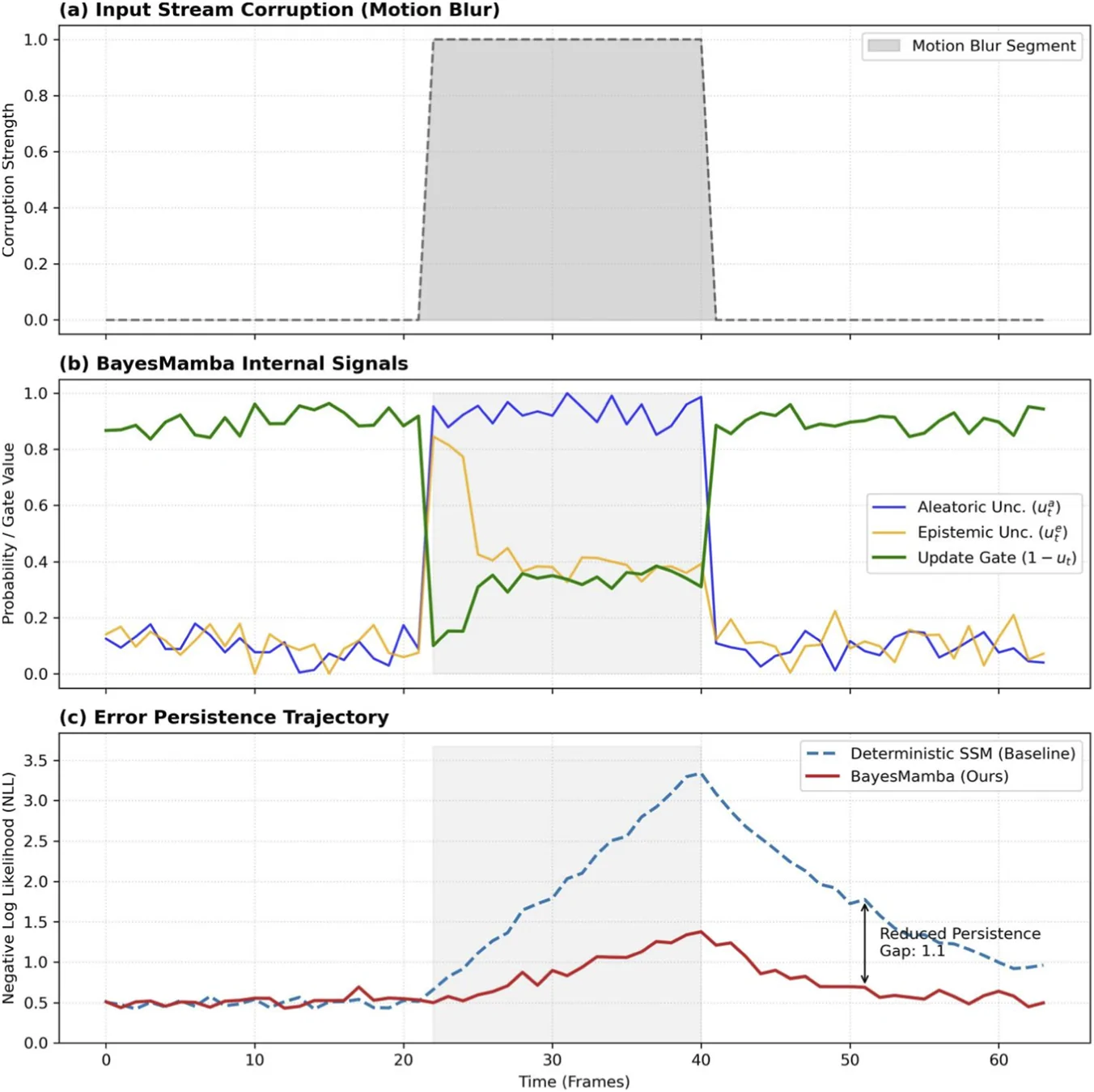
Qualitative example of uncertainty and error persistence under motion blur. **(a)** The shaded region marks a motion-blur corruption interval in the input stream. **(b)** BayesMamba internal signals show aleatoric uncertainty, epistemic uncertainty, and the update gate during the corrupted segment. **(c)** The error persistence trajectory compares BayesMamba with the deterministic baseline and shows a reduced persistence gap after the corrupted interval.

For completeness, Table 5 summarized the corruption suite, Table 2 listed hyperparameters, and Table 3 reported additional evaluation on challenging subsets. Figure 7 provided qualitative examples.

Figure 7 provided an illustration of the proposed mechanism on a blur-corrupted segment, showing how uncertainty rose during unreliability and how B-Scan suppressed state updates to reduce error persistence.

##### 3.3.9.1 Persistence evaluation protocol

(1) Corrupted segments of length Lcorr = 0.3T are placed at the middle of each video [tstart = 0.35T, tend = 0.65T]. (2) Frame-level predictions are obtained under online streaming mode, where each prediction is based only on historical context. (3) Parameter τ determines the post-corruption evaluation window W = {tend+1,,min (tend+τ,T)}. Videos with |W|<16 are filtered. (4) Category balancing: 10 videos per action category (1,740 total). (5) B = 10,000 bootstrap resampling runs for confidence intervals. BayesMamba reduced Persist (64) from 0.41 to 0.19 and Persist (128) from 0.38 to 0.16.

## 4 Discussion

From the empirical analysis one can see the core hypothesis of the paper: uncertainty-guided discretization can preserve the linear-time advantage of selective SSMs while reducing the degree of errors after contamination. BayesMamba injects the reliability signal into the discretization parameters, causing uncertainty to affect how memory is updated, rather than being simple prediction calibration.

### 4.1 Reliability-aware discretization and persistence

The persistence metric represents a failure mode that the accuracy of a contaminated segment may ignore: a short unreliable segment can bias the internal state and continue to produce errors after the segment ends. Under bursty motion blur on SSv2, BayesMamba significantly reduced persistence (Persist (64): 0.41 -> 0.19; Persist (128): 0.38 -> 0.16), and improved robust accuracy (Table 1). When uncertainty rises, B-Scan suppresses state updates, thereby limiting unreliable evidence from being injected into the state.

### 4.2 Calibration and state dynamics

Temperature scaling improves confidence calibration through logits (Salman et al., 2020), but it does not modify the temporal update rule. This separation is very obvious: it improves calibration (Table 11) and keeps the accuracy of the deterministic baseline (Table 4), yet it does not bring corresponding robustness gains (Table 1). BayesMamba improves calibration and robustness under distribution shift. Its reliability signal acting on the mechanism that transfers information across time is very effective.

### 4.3 Aleatoric and epistemic signals

Qualitative trajectories (Figures 2, 7) show that uncertainty is temporally localized. This characteristic is consistent with the bursty properties of motion blur and short attacks. Through Aleatoric/Epistemic decomposition analysis, sensor-like noise raises aleatoric uncertainty, and adversarial perturbations raise epistemic uncertainty (Kendall and Gal, 2017; Gal and Ghahramani, 2016; Lakshminarayanan et al., 2017). Related methods are also used for uncertainty-based spatiotemporal attention (Guo et al., 2022). The unique aspect of BayesMamba is that it directly integrates uncertainty into the discretization process of the SSM.

### 4.4 Limitations

BayesMamba relies on uncertainty estimation (Table 2), which introduces additional computation during inference. Although Table 8 and Figure 6 show that the additional overhead is small under the empirical setting of this paper, practical latency requirements may still require reducing S and accepting weaker uncertainty estimates. The adversarial evaluation mainly focuses on FGSM/PGD under an ℓ ∞ budget. If adaptive attackers attack the uncertainty head or discretization parameters, the current gains may be weakened. The robustness in this paper is not certified robustness. The empirical study uses benchmark datasets and predefined protocols, and broader validation is still needed on more real-world data and more failure modes.

### 4.5 Broad impact

Robust long-horizon action recognition can reduce persistent errors after momentary sensor failures and improve the safety of assistive systems. When this type of model is applied to surveillance scenarios, reporting robustness, persistence, and calibration can reduce overconfident deployment decisions. We must also consider the necessity of policy and privacy safeguards.

### 4.6 Conclusion

This work examines whether uncertainty-guided discretization can reduce the persistence of errors after contamination and improve robustness without sacrificing linear-time inference capability in long-horizon action recognition. It proposes BayesMamba and Bayesian Selective Scan (B-Scan), which integrate Bayesian uncertainty into the discretization of selective SSMs by modulating the effective time step and reliability-modulated input injection.

On Kinetics-400 and Something-Something V2, BayesMamba keeps relatively small computational overhead compared with deterministic selective SSMs and retains accuracy on clean data (Table 4). On SSv2, BayesMamba achieves stable robustness improvements under bursty motion blur and PGD, and reduces error persistence after the contaminated segment ends (Table 1). BayesMamba also improves calibration under distribution shift (Table 11), showing that reliability-aware state updates can be used together with post hoc calibration methods.

Future work includes evaluating stronger adaptive attack models targeting uncertainty pathways, extending persistence evaluation to more long video stream benchmarks and real-world streaming scenarios, and exploring alternative uncertainty estimation methods that can reduce stochastic overhead while preserving reliability-aware dynamics.

## Data Availability

Publicly available datasets were analyzed in this study. The Kinetics-400 dataset is available under a CC-BY 4.0 license. The Something-Something V2 dataset is available for research use.
